# Work and mental health in doctors: A short review of Norwegian studies

**DOI:** 10.1097/j.pbj.0000000000000050

**Published:** 2019-09-09

**Authors:** Reidar Tyssen

**Affiliations:** Department of Behavioral Sciences in Medicine, Faculty of Medicine, Institute of Basic Medical Sciences, University of Oslo, Oslo, Norway

Previous studies have found relatively good physical health in doctors, whereas several studies now report relatively high levels of mental distress among them.^1–5^ This applies in particular to stress, burnout, and depressive symptoms—and especially among medical students and young doctors early in their careers. However, we lack representative prevalence studies of mental disorders among doctors. There is little empirical support for the notion that there is more mental distress in medical students compared to that in other university students, nor do they differ from other students with respect to personality traits.^6^

Despite this, several studies have found more suicide among physicians than in other occupational groups.^7,8^ This may be partly due to their attempts in committing suicide being more frequently successful; yet, this may also represent the tip of an iceberg of frustration and inadequate mental health care among medical doctors.^9^

## Presumed risk factors from longitudinal studies

What do we know about individual and work-related predictors and risk factors of mental distress from the prospective and longitudinal studies so far? Some landmark early follow-up studies in the United States and United Kingdom put doctors’ work and mental health on the agenda in the 1970s and 1980s.^10–12^ In the following, we will pay most attention to the Longitudinal Study of Norwegian Medical Students and Doctors (NORDOC).^13,14^ This study has since 1993/1994 followed repeatedly 2 cohorts of medical students (N = 1052) with 6 years apart for 20 years (2014), and there is now an ongoing 25-year follow up. For more information, see Facebook: @docsinrush.

There are 2 main hypotheses with regard to possible risks factors. First, it may be due to individual factors such as personality traits, past mental health problems, etc. Second, contextual stress may influence mental health among doctors, whether this is unhealthy working conditions or negative life events (ie, stress outside of work). Both individual and work-related factors seem to be of importance. Individual factors may be more important with respect to more severe clinical mental disorders, whereas work-related factors are more important for stress, burnout, and minor emotional disturbance.^5,15^

In terms of individual factors, NORDOC has included personality traits, as one of very few studies in doctors. Neuroticism personality trait is related to vulnerability, self-criticism, low self-esteem, and proneness to stress compatible with the modern common term “hypersensitivity.” This trait predicts stress, anxiety, and depression in the general population,^16^ and, as expected, in NORDOC it predicts work stress, burnout, and even severe depressive symptoms among doctors.^14,17,18^ Studies among medical students and young doctors have found the combination of conscientiousness (or obsessiveness) and neuroticism seems to be especially important for school and work stress.^19,20^ In addition, NORDOC has identified a particular trait (reality weakness) that is associated with severe personality pathology.^6^ This trait predicts independently a need for mental health treatment,^21^ lack of help-seeking,^22^ severe depressive symptoms,^18^ and even aggravation of suicidal ideation among medical students and doctors.^23^ Another important individual factor is the increased rate of female medical students and young doctors. In Norway, there has been an increase from 55% to 70% of women in medical schools during the past 2 decades. We have previously found little gender differences in NORDOC, but a recent study among Norwegian medical students find considerable reduction in subjective well-being in 2015 compared to that 20 years ago, and this reduction was most prominent among the female students.^24^ This reflects recent trends in Norway and other Western societies which observe increased anxiety and depressive symptoms among young female adults.^25,26^

With regard to contextual stress, it seems that both work-related stress and stress outside of work are of importance. NORDOC studies have found that demanding patient work is associated with mental health problems early in the medical career,^27^ and that difficulty with balancing life—such as work–home interface stress—is a sustaining problem over the course of the career.^28^ The detrimental role of such stress is also in keeping with studies among US doctors.^5^ Work–home stress predicts burnout (emotional exhaustion) in a NORDOC 5-year follow-up study.^29^ A promising finding is that such stress was less prominent in the youngest cohort of Norwegian doctors 10 years after leaving medical school.^30^ This may be due to increased coverage of kindergarten as well as changed and more liberal gender roles in our Scandinavian society over recent years.

There are also studies that associate time pressures and burnout with suicidal ideation among medical students and doctors.^31,32^ Sleep-deprivation due to call work and long hours may be one important reason for more depressive symptoms measured in young doctors.^2^ A recent NORDOC study of life satisfaction during 15 years of the career controlled for all possible individual factors, and found the following work-related predictors and possible risk factors: work–home stress, lack of colleague support, and emotional demands at work.^33^ Doctors often feel a 24/7 responsibility and obligation for individual patients and their treatment and this puts extraordinary emotional demands on this occupational group.

## Does stress among doctors have consequences for their patient care?

Many studies can indicate lowered quality of patient care among stressed doctors with burnout, but a large majority of these studies build on self-report by the doctors themselves of more errors and poorer care.^5,34^ We lack an empirical foundation for the notion that stress and burnout really impair doctors’ functioning with respect to observed poorer quality of care. There are 2 classical observation studies demonstrating that long hours and time pressures interfere with doctors functioning,^35,36^ but we lack studies that find burnout to lead to observed errors or poorer care.^37^ The burnout concept and scales are not very valid with respect to impaired functioning, for example, with respect to valid cut-off for defining a case.^38^ On the other hand, depression and other mental disorders lead to poor functioning.^39^ We need more studies on working conditions and the levels of stress and poor health among young doctors that lead to lowered patient care.

## What are the most common mental disorders among doctors?

In general, doctors may have the same disorders that strike anyone else; doctors are not invincible. Although depressive symptoms seem to be prevalent in the early years of the medical career, some of this may be due to exhausting work stress by frequent on-call work.^40^ We lack representative studies on the occurrence of valid depression among doctors compared to that in other occupational groups. Suicide is more common among doctors than among other groups of academics, but since it is also very common in veterinarians, this may also be due to available knowledge and means (drugs) for committing suicide during mental health deterioration.^41^ Alcoholism and drug abuse is an additional known risk factor for suicide and the SAD triad (suicidal behavior–alcoholism–depression) may be particularly important for medical doctors.^42^ From clinical experience with doctor–patients, we know the slippery slope from self-medication with tranquilizers to cope with the stresses to dependency of alcohol and drugs, in addition to other boundary violations.^43^ There are very few clinical studies including diagnostic interviews among doctors. One previous Spanish study emphasizes the importance of dual diagnoses, especially in alcohol dependence and mood disorders.^44^ From own experience, we know that bipolar disorder (type II) is quite common among physicians, but we lack sound empirical studies that compare occurrence of mental disorders in doctors with that in other groups. American impaired physician programs have for many years shown high and promising recovery rates (70–80%).^4^ The programs used to focus on addiction and substance abuse, but they now put increasing emphasis on psychiatric diagnoses. A family history, opioid use, and psychiatric comorbidity predicted relapse of substance abuse among doctors and other healthcare workers.^45^

In Norway, we have implemented a successful low-threshold intervention, the Villa Sana program.^17^ This intervention seems to reduce burnout in doctors. It includes 2 separate schemes, a 1-day individual counseling scheme, and a 1-week group-based scheme in a psychiatric hospital. The Norwegian Medical Association pays for the program that is free for all doctors.

With respect to medical students and young doctors, we have also a large longitudinal study on mindfulness-based stress reduction.^46^ This is a randomized-controlled trial of second year medical and psychology students, and they have now been followed-up for 6 years, for the medical students into the first 2 postgraduate years.^47^ The reduction of emotional distress by mindfulness training is most prominent in female students.^46^ The training has a stronger impact among those with vulnerable personality (high neuroticism and conscientiousness).^48^ During the follow-up, there is an increase in active coping and reduction in passive or avoidance coping—the effects on ways of coping may be important psychological mechanisms of mindfulness training.^47^

## Future research challenges

We need more long-term follow-up studies that use validated instruments to capture changes in working conditions and their impact on physician health. For instance, there are few studies in doctors of Karasek's Demand-Control model.^49^ There are more studies by this model in other healthcare workers. More studies are required that measure the effect of physicians’ health problems on their performance and patient care. Gender issues are important, since there are now more women entering the medical career. As mentioned, we also need more studies with diagnostic interviews that compare frequency of valid disorders in samples of physicians with that in other groups. Doctors are nowadays moving, and we should study the effect of globalization on doctor's health. Cross-national disparities may be due to differences in the health systems, working conditions, etc.^50,51^ Finally, we need more studies on positive psychology and factors that may promote and enhance well-being among physicians.

## Acknowledgments

Robert Scott Findlay is gratefully acknowledged for English language proof reading.

## Conflicts of interest

The author declare no conflicts of interest.
